# Pulse transit time-estimated blood pressure: a comparison of beat-to-beat and intermittent measurement

**DOI:** 10.1038/s41440-022-00899-z

**Published:** 2022-04-06

**Authors:** Satoshi Hoshide, Akiomi Yoshihisa, Fumihiro Tsuchida, Hiroyuki Mizuno, Hiroki Teragawa, Takatoshi Kasai, Hitoshi Koito, Shin-ichi Ando, Yoshihiko Watanabe, Yasuchika Takeishi, Kazuomi Kario

**Affiliations:** 1 Division of Cardiovascular Medicine, Jichi Medical University School of Medicine, Tochigi, Japan; 2 Department of Cardiovascular Medicine, Fukushima Medical University, Fukushima, Japan; 3 Department of Clinical Laboratory Sciences, Fukushima Medical University School of Health Sicence, Fukushima, Japan; 4 Department of Pulmonary Medicine, Yabuki Hospital, Yamagata, Japan; 5 Department of Cardiovascular Medicine, JR Hiroshima Hospital, Hiroshima, Japan; 6 Cardiovascular Respiratory Sleep Medicine, Department of Cardiovascular Medicine, Juntendo University Graduate School of Medicine, Tokyo, Japan; 7 Department of Internal Medicine, Misugikai Otokoyama Hospital, Kyoto, Japan; 8 Sleep Apnea Center, Kyushu University Hospital, Fukuoka, Japan; 9 Department of Internal Medicine, Nippon Dental University Hospital, Tokyo, Japan

**Keywords:** Pulse transit time, Beat-to-beat, Blood pressure variability, Continuous blood pressure monitoring

## Abstract

Pulse transit time (PTT), which refers to the travel time between two arterial sites within the same cardiac cycle, has been developed as a novel cuffless form of continuous blood pressure (BP) monitoring. The aim of this study was to investigate differences in BP parameters, including BP variability, between those assessed by beat-to-beat PTT-estimated BP (eBP_BTB_) and those assessed by intermittent PTT-estimated BP at fixed time intervals (eBP_INT_) in patients suspected of having sleep disordered breathing (SDB). In 330 patients with SDB (average age, 66.8 ± 11.9 years; 3% oxygen desaturation index [ODI], 21.0 ± 15.0/h) from 8 institutes, PTT-estimated BP was continuously recorded during the nighttime. The average systolic eBP_BTB_, maximum systolic and diastolic eBP_BTB_, standard deviation (SD) of systolic and diastolic eBP_BTB_, and coefficient variation (CV) of systolic and diastolic eBP_BTB_ were higher than the respective values of eBP_INT_ (all *P* < 0.05). Bland–Altman analysis showed a close agreement between eBP_BTB_ and eBP_INT_ in average systolic BP and SD and CV of systolic BP, while there were disagreements in both minimum and maximum values of eBP_BTB_ and eBP_INT_ in patients with high systolic BP (*P* < 0.05). Although systolic BP variability incrementally increased according to the tertiles of 3%ODI in both eBP_BTB_ and eBP_INT_ (all *P* < 0.05), there was no difference in this tendency between eBP_BTB_ and eBP_INT_. In patients with suspected SDB, the difference between eBP_BTB_ and eBP_INT_ was minimal, and there were disagreements regarding both the minimum and maximum BP. However, there were agreements in regard to the index of BP variability between eBP_BTB_ and eBP_INT_.

## Graphical abstract

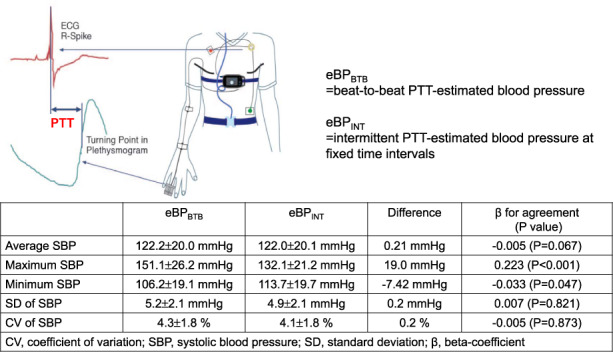

## Introduction

Blood pressure (BP) fluctuates over time. However, a precise assessment of BP variability is only possible with beat-to-beat BP recordings using a specific methodology, such as intra-arterial BP monitoring or continuous finger BP measurements [1, 2]. Increases in BP variability can be observed across various timeframes [3, 4]. Over a short time period, ambulatory BP monitoring (ABPM) using a device that intermittently measures BP at fixed time intervals is widely used and has allowed for the estimation of BP variability, which has been shown to be associated with target organ damage and cardiovascular prognosis [5–9].

Pulse transit time (PTT), which refers to the travel time of the systolic pressure wave between two arterial sites, typically the aortic valve and a peripheral site, has been developed as a novel cuffless form of continuous BP monitoring [10–12]. Several previous studies reported that the PTT-estimated BP value was validated by the BP value evaluated by a mercury sphygmomanometer [10, 11]. The BP variability assessed by beat-to-beat PTT-estimated BP may detect BP variability to a greater extent than ABPM in situations of dramatic BP change. However, regarding the methodology of BP evaluation using PTT, there has been no study on whether transient BP elevation and fluctuation assessed by beat-to-beat PTT-estimated BP provides a clinical advantage compared to that assessed by intermittent BP readings at fixed time intervals.

In sleep disorder breathing (SDB), acute transient BP elevation and fluctuation are observed at the end of desaturation episodes during sleep [4, 13–17]. We hypothesized that BP readings assessed by beat-to-beat PTT-estimated BP variability (eBP_BTB_) would be a more precise measure of BP variability than those assessed by intermittent PTT-estimated BP variability at a fixed time interval (eBP_INT_) in patients with SDB. The aim of this study was to investigate the difference in BP parameters, including BP variability, between those assessed by eBP_BTB_ and those assessed by eBP_INT_ in patients suspected of SDB from a multicenter study.

## Subjects and methods

### Subjects

The subjects enrolled in this study were analyzed using a SOMNO touch RESP (Fukuda Denshi Co., LTd., Tokyo, Japan), which recorded nasal airflow, snoring sounds, thoracic and abdominal respiratory effort signals, ECG, oxygen saturation (SpO2) via pulse oximetry, PTT, R-R timing, finger plethysmography and body position. The subjects comprised 330 patients suspected of having SDB at 8 institutes (Supplementary Material) between May 2016 and August 2019. All recordings were analyzed for the oxygen desaturation index (ODI) using DOMINO Light software version 1.5.0 (Somnomedics, Randersacker, Germany). The 3% ODI was calculated as the number of oxygen desaturation events (reduction of 3% from baseline) per hour during the entire recording time. The Institutional Review Board of Jichi Medical University approved the study with a waiver of informed consent.

Demographic information was collected by physicians at each participating institute. Diagnosed hypertension, dyslipidemia and diabetes were defined as a self-reported physician’s diagnosis or current use of the respective treatment medication(s). Body mass index (BMI) was calculated from measured weight and height. Office BP measurements were obtained at local medical centers using validated cuff oscillometric devices.

### Determinant of blood pressure by pulse transit time

Several previous studies have reported validation of the determination of BP by PTT [11]. In brief, PTT is defined as the travel time between the R-wave of the ECG and the pulse wave at the site of the finger in plethysmography (Supplementary Fig. 1). Arrival refers to the steepest part of the leading edge of the pulse wave. Pulse wave velocity was determined by PTT, height, and body composition factors [10, 18]. Systolic BP (SBP) and diastolic BP (DBP) values determined by PTT were calculated automatically using DOMINO Light software version 1.5.0 based on a patented algorithm (11/364174 US 2006/0217616 A1, 7374542). Detailed information for the calculation of beat-to-beat PTT-estimated BP is shown in the Supplementary Material. Resting BP, which was measured just before placing SOMNO touch RESP on patients in the supine position, was used for calibration by a manual, cuff-based method. All BP readings estimated by PTT from the start to the end of the SOMNO touch RESP recording were used to calculate average systolic and diastolic eBP_BTB_ values. eBP_INT_ was defined every 30 min during the recording, and average systolic and diastolic eBP_INT_ values were calculated from those values. The maximum values of eBP_BTB_ and eBP_INT_ were defined as the highest values among all BP values of eBP_BTB_ and eBP_INT_, respectively. The minimum values of eBP_BTB_ and eBP_INT_ were defined as the lowest values among all BP values of eBP_BTB_ and eBP_INT_, respectively. For BP variability indices, we calculated 1) the standard deviation (SD) of the average eBP_BTB_ and eBP_INT_ and 2) the coefficient of variation (CV) of the average eBP_BTB_ and eBP_INT_.

### Statistical analysis

Data are expressed as means ±standard deviations or percentages. A two-tailed paired *t* test was used to compare the mean BP indices between eBP_BTB_ and eBP_INT_. To assess the agreement between eBP_BTB_ and eBP_FIX_, we performed Bland–Altman analysis. The relationships between eBP_BTB_ and eBP_FIX_ are shown in Bland–Altman plots and were examined by linear regression analysis. Moreover, linear regression analysis was used for the association between eBP_BTB_ and eBP_INT_ parameters, their differences and tertiles of ODI. A two-sided p value **<**0.05 was accepted as significant. All statistical analyses were performed with Stata ver. 15.0 software (StataCorp, College Station, TX, USA).

## Results

Table 1 provides the demographic variables and clinical characteristics of the included patients. The average age was 66.8 ±11.9 years. The proportion of patients with heart failure was relatively high (45.8%). The average ODI was 21.0 ±15.0/h.

**Table 1:** Patient characteristics (*n* = 330)

Age, years	66.8 ± 11.9
Male, %	68.5
BMI, kg/m^2^	24.3 ± 4.4
Hypertension, %	65.8
Diabetes, %	33.3
Dyslipidemia, %	63.6
Atrial fibrillation, %	35.2
Prevalent CAD, %	26.4
Prevalent stroke, %	9.1
Prevalent heart failure, %	45.8
Anti-hypertensive drug	
Calcium blocker, %	37.0
Angiotensin II receptor blocker, %	29.1
ACE inhibitor, %	26.1
Diuretics, %	34.2
Beta blocker, %	55.8
Alfa blocker, %	1.2
Office SBP, mmHg	128.0 ± 21.6
Office DBP, mmHg	74.4 ± 16.2
ODI, per 1 h	21.0 ± 15.0

Data are means ±SDs or percentages

*ACE* angiotensin-converting enzyme, *BMI* body mass index, *CAD* coronary artery disease, *DBP* diastolic blood pressure, *ODI* oxygen desaturation index, *SBP* systolic blood pressure

**Fig. 1:**
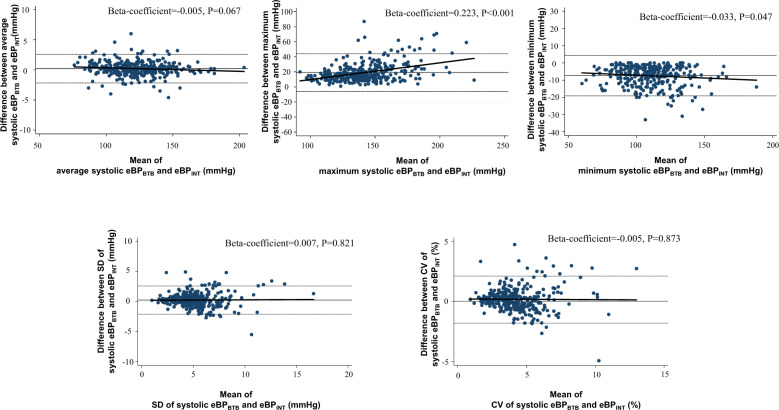
Bland–Altman plots comparing systolic blood pressure parameters between eBP_BTB_ with eBP_INT_. eBP_BTB_ indicates beat-to-beat PTT-estimated BP, eBP_INT_ intermittent PTT-estimated BP at fixed time intervals

Table 2 shows the comparison of each BP index between eBP_BTB_ and eBP_INT_. Except for diastolic eBP_BTB_ and eBP_INT_, statistically significant differences in BP indices were found between eBP_BTB_ and eBP_INT_ values. The average systolic eBP_BTB_, maximum systolic and diastolic eBP_BTB_, Standard deviation (SD) of systolic and diastolic eBP_BTB_, and CV of systolic and diastolic eBP_BTB_ were higher than those of eBP_INT_. Bland–Altman analysis demonstrated a closer agreement between eBP_BTB_ and eBP_INT_ for average SBP, SD of SBP and CV of SBP (i.e., BP variability measures), while there were significant disagreements between both minimum and maximum values of eBP_BTB_ and eBP_INT_ in patients with high SBP (Fig. 1). We performed a stratified analysis according to the presence and absence of atrial fibrillation. In Bland–Altman analysis, there was significant disagreement between the maximum value of systolic eBP_BTB_ and eBP_INT_ irrespective of the presence or absence of atrial fibrillation (Supplementary Figs. 2 and 3). In addition, compared to the difference in eBP_BTB_ and eBP_INT_ between the group with atrial fibrillation and that without, there were no significant differences in any of the BP indices (Supplementary Table 1). Supplementary Tables 2–6 show a comparison of BP indices in eBP_BTB_ and eBP_INT_ according to the use of each antihypertensive drug. The group using calcium channel blockers exhibited a greater difference between eBP_BTB_ and eBP_INT_ in maximum SBP and minimum DBP than the group without calcium channel blockers. In contrast, the group treated with ACE inhibitors, diuretics, or beta-blockers showed a smaller difference between eBP_BTB_ and eBP_INT_ in some BP indices.

**Table 2:** Comparison of pulse transit time (PTT)-estimated blood pressure parameters between beat-to-beat and intermittent measurement

	Beat-to-beat measurement	Intermittent measurement	Difference
Average SBP, mmHg	122.2 ± 20.0	122.0 ± 20.1	0.21**
Average DBP, mmHg	71.9 ± 13.7	71.9 ± 13.7	−0.01
Maximum SBP, mmHg	151.1 ± 26.2	132.1 ± 21.2	19.0***
Maximum DBP, mmHg	86.5 ± 14.0	78.6 ± 13.8	7.8***
Minimum SBP, mmHg	106.2 ± 19.1	113.7 ± 19.7	−7.42***
Minimum DBP, mmHg	58.8 ± 15.0	65.2 ± 14.3	−6.5***
SD of SBP, mmHg	5.2 ± 2.1	4.9 ± 2.1	0.2***
SD of DBP, mmHg	3.7 ± 1.3	3.6 ± 1.5	0.1*
CV of SBP, %	4.3 ± 1.8	4.1 ± 1.8	0.2***
CV of DBP, %	5.4 ± 2.8	5.3 ± 2.9	0.2*

*CV* coefficient variation, *DBP* diastolic blood pressure, *SBP* systolic blood pressure, *SD* standard deviation.

^*^P < 0.05; **P < 0.01; ***P < 0.001 between groups

Table 3 compares the systolic BP indices of eBP_BTB_ and eBP_INT_ according to the tertiles of 3% ODI. In both eBP_BTB_ and eBP_INT_, the SD and CV of both systolic eBP_BTB_ and eBP_INT_ incrementally increased according to the tertiles of 3% ODI. However, there was no interaction between BP indices and tertiles of 3% ODI according to the difference in eBP_BTB_ and eBP_INT_. These associations were observed in the diastolic BP index between eBP_BTB_ and eBP_INT_ according to the tertiles of 3% ODI (Supplementary Table 7).

**Table 3:** Comparison of pulse transit time (PTT)-estimated systolic blood pressure parameters between beat-to-beat and intermittent measurement according to tertiles of ODI

	ODI/h			*P* for trend	*P*_int_
	Tertile 1 (0–11.3) *n* = 110	Tertile 2 (11.4–24.9) *n* = 110	Tertile 3 (25.1–74.4) *n* = 110		
Average SBP, mmHg					
Beat-to-beat	122.5 ± 17.2	119.9 ± 20.3	124.2 ± 22.1	0.524	0.985
Intermittent	122.3 ± 17.1	119.7 ± 20.5	124.0 ± 22.4	0.544	
Difference	0.17 ± 1.27	0.23 ± 1.06^*^	0.25 ± 1.24^*^	0.651	NA
Maximum SBP, mmHg					
Beat-to-beat	150.2 ± 23.5	147.9 ± 25.0	155.4 ± 29.2	0.142	0.699
Intermittent	131.8 ± 17.9	129.4 ± 21.0	135.2 ± 24.0	0.230	
Difference	18.4 ± 13.6^‡^	18.5 ± 12.3^‡^	20.2 ± 11.6^‡^	0.300	NA
Minimum SBP, mmHg					
Beat-to-beat	107.2 ± 16.8	104.2 ± 19.9	107.4 ± 20.5	0.930	0.910
Intermittent	114.4 ± 17.0	111.5 ± 20.2	115.1 ± 21.7	0.809	
Difference	−7.3 ± 6.0^‡^	−7.3 ± 5.7^‡^	−7.7 ± 5.9^‡^	0.598	NA
SD of SBP, mmHg					
Beat-to-beat	4.8 ± 1.6	5.1 ± 2.1	5.6 ± 2.4	0.006	0.934
Intermittent	4.7 ± 1.8	4.7 ± 2.0	5.4 ± 2.3	0.008	
Difference	0.17 ± 1.32	0.31 ± 1.00^†^	0.20 ± 1.17	0.836	NA
CV of SBP, %					
Beat-to-beat	4.0 ± 1.3	4.3 ± 2.0	4.6 ± 1.9	0.021	0.827
Intermittent	3.9 ± 1.6	4.0 ± 1.8	4.4 ± 1.8	0.009	
Difference	0.12 ± 1.10	0.27 ± 0.88^†^	0.14 ± 0.93	0.858	NA

*P*_int_ mean *P* for interaction between beat-to-beat and intermittent group

*CV* coefficient of variation, *SBP* systolic blood pressure, *SD* standard deviation, *ODI* oxygen desaturation index, *NA* not applicable

^*^P < 0.05; ^†^*P* < 0.01; ^‡^*P* < 0.001 between the value of beat-to-beat and intermittent PTT-estimated BP values in each tertile

## Discussion

This study performed a comparison of PTT-estimated BP indices estimated by beat-to-beat and intermittent measurements in patients with suspected SDB. The results can be summarized as follows. First, although there was a statistically significant difference between average systolic eBP_BTB_ and average systolic eBP_INT_, the absolute difference was minimal. Second, there were significant disagreements in both minimum and maximum systolic BP between the eBP_BTB_ and eBP_INT_ methods, while there was a close agreement in BP variability between the systolic eBP_BTB_ and systolic eBP_INT_. Third, BP variability incrementally increased according to the tertiles of 3% ODI in both systolic eBP_BTB_ and systolic eBP_INT_, and this tendency was essentially the same for systolic eBP_BTB_ and systolic eBP_INT_.

To detect BP fluctuation over a certain period, continuous BP reading is ideal. However, percutaneous implantation of a monitor in the radial artery is the only established method, and among the numerous approaches that have been proposed as alternatives, ABPM is the closest to percutaneous implantation in the radial artery. BP readings measured by ABPM have been widely used to evaluate the effect of antihypertensive drugs and have been shown to be associated with target organ damage and cardiovascular events [19, 20]. Approximately 40 years ago, di Rienzo et al. compared the data of continuous, 24-h monitoring of each pressure wave and single pressure waves taken at regular intervals of 5, 10, 15, 30, and 60 min using invasive BP measurement [21]. Their results showed almost no difference in average SBP between the continuous measurement of each wave and the measurement of single waves at intervals if the interval was within 30 min. Thus, a previous study showed that the appropriate interval is available to obtain average BP readings comparable to continuous BP readings during a certain period assessed by ABPM.

However, this study did reveal the possibility that minimum and maximum BP values evaluated every 30 min are different from beat-to-beat values. Intermittent BP measurement is not able to detect extreme, anomalous BP changes. In clinical practice, detecting the maximum BP value during the nighttime may be important for patients with SDB. Previously, using a noninvasive oscillometric, desaturation-triggered BP measurement device, we found that cases with recurrent stroke and obstructive sleep apnea occasionally registered SBP readings above 200 mmHg even though the average nighttime SBP taken at intermittent intervals was 167 mmHg [22].

In this study, the group using calcium channel blockers exhibited a greater difference between eBP_BTB_ and eBP_INT_ in maximum SBP and minimum DBP than the group that did not use calcium channel blockers. BP variability has been strongly correlated with BP level [23]. In this study, the group that used calcium channel blockers exhibited higher eBP_BTB_ and eBP_INT_ in average SBP and DBP than those that did not use calcium channel blockers. Therefore, the group using calcium channel blockers may exhibit a larger difference between eBP_BTB_ and eBP_INT_ in some BP indices than the group that did not use calcium channel blockers. In contrast, the group treated with ACE inhibitors, diuretics, or beta-blockers showed a smaller difference between eBP_BTB_ and eBP_INT_ in some BP indices. This finding may have been attributable to the patient characteristics in this study, since ~50% of our enrolled patients had prevalent heart failure. These drugs are mainly used in patients with heart failure. Since heart failure involves low cardiac output and autonomic nervous dysfunction, heart failure patients demonstrate a lesser BP response to environmental stimulation.

In this study, BP variability assessed by systolic eBP_BTB_ incrementally increased across the tertiles of ODI. A previous study reported that there is a linear association between systolic eBP_BTB_ and ODI in 242 patients with suspected SDB [24]. Although the results of this study confirmed those of a previous study, there was no interaction of this association with BP variability assessed by systolic BP_INT_. There have been several previous studies about the association between BP variability assessed by fixed-interval BP measurement using ABPM and the severity of SDB [25, 26]. Steinhorst AP et al. showed that patients with an apnea hypopnea index (AHI) ≥10 had higher nighttime BP variability assessed by ABPM at 20-min intervals than those without, while this association was not found in daytime BP variability [25]. Therefore, fixed-interval BP measurement is useful for detecting nighttime BP variability due to SDB. Interestingly, a previous study reported that when the interval of BP measurement was longer, BP variability evaluated at fixed time intervals was higher than that of beat-to-beat BP measurement [21]. This potential error caused by sampling size may mask the difference between BP variability assessed by systolic eBP_BTB_ and by systolic eBP_INT_. Regardless of the measurement method, BP variability is expected to increase according to the severity of SDB.

There are important limitations in this study. We acknowledge that there are currently insufficient data to conclude that the PTT-estimated BP value is comparable to the BP value taken by conventional BP measurement techniques, even though the PTT-estimated BP value has been validated with a mercury sphygmomanometer according to the international validation protocol [11]. PTT is calculated with a formula using PWV, which is influenced by many factors, such as age, sex, arterial elastic properties, and respiratory status. In addition, although there is little difference between BP assessed by conventional BP measurement and PTT-estimated BP at rest, there is a large difference of ~20 mmHg between them during exercise [10]. Therefore, in this study, we assessed PTT-estimated BP at night, when the effect of exercise was less pronounced. Furthermore, a previous study indicated that PTT-estimated BP can be used for the assessment of relative BP changes within the same individuals [27]. As a result, international BP guidelines have not yet accepted BP measurement using a wearable device, including the PTT methodology for diagnostic and treatment decisions [28]. The clinical importance of PTT-estimated BP and whether BP indices estimated by PTT are associated with the risk of target organ damage or cardiovascular events more than conventional BP measurements, such as in-office and out-of-office BP measurements, require further research.

In conclusion, this study showed that the PTT-estimated BP difference between beat-to-beat and intermittent measurements was minimal in patients with suspected SDB. In addition, there were disagreements in both minimum and maximum BP between PTT estimated by beat-to-beat monitoring and that estimated by intermittent measurement, while no disagreement was observed in the index of BP variability. The advantage of continuous BP monitoring might be the ability to identify maximum and minimal BPs, the clinical implications of which should be clarified in the future.

## Supplementary information

Supplementary information
