# Supplementary material for: Pulse transit time-estimated blood pressure: a comparison of beat-to-beat and intermittent measurement
Source: Hypertens Res. 2022 Apr 6;45(6):1001–7. doi: 10.1038/s41440-022-00899-z (PMC9106575; doi:10.1038/s41440-022-00899-z)
Supplement: Supplementary file 1 — Supplementary information [file 41440_2022_899_MOESM1_ESM.docx]

*Supplemental file*

**Pulse Transit Time-Estimated Blood Pressure: A Comparison of**

**Beat-to-Beat and Intermittent Measurement**

Satoshi Hoshide^1^, Akiomi Yoshihisa^2,3^, Fumihiro Tsuchida^4^, Hiroyuki Mizuno^1^, Hiroki Teragawa^5^, Takatoshi Kasai^6^, Hitoshi Koito^7^, Shin-ichi Ando^8^, Yoshihiko Watanabe^9^, Yasuchika Takeishi^2^, Kazuomi Kario^1^

^1^Division of Cardiovascular Medicine, Jichi Medical University School of Medicine

^2^Department of Cardiovascular Medicine, Fukushima Medical University

^3^Department of Clinical Laboratory Sciences, Fukushima Medical University School of Health Sicence

^4^ Department of Pulmonary Medicine, Yabuki Hospital

^5^ Department of Cardiovascular Medicine, JR Hiroshima Hospital

^6^ Cardiovascular Respiratory Sleep Medicine, Department of Cardiovascular Medicine, Juntendo University Graduate School of Medicine

^7^ Department of Internal Medicine, Misugikai Otokoyam Hospital

^8^ Sleep Apnea Center, Kyushu University Hospital

^9^ Department of Internal Medicine, Nippon Dental University Hospital

**Correspondence:** Dr. Kazuomi Kario, MD, PhD, Division of Cardiovascular Medicine, Department of Medicine, Jichi Medical University School of Medicine, 3311-1 Yakushiji, Shimotsuke, Tochigi 329-0498, Japan.

Tel.: +81-285-58-7538, Fax: +81-285-44-4311.

Email: kkario@jichi.ac.jp

**Participants and participating centers**

Satoshi Hoshide: Jichi Medical University School of Medicine; Akiomi Yoshihisa: Fukushima Medical University; Fumihiro Tsuchida: Yabuki Hospital; Hiroyuki Mizuno: Jichi Medical University School of Medicine; Teragawa Hiroki: JR Hiroshima Hospital; Takatoshi Kasai: Juntendo University Graduate School of Medicine; Hitoshi Koito: Otokoyama Hospital; Shin-ichi Ando: Kyushu University Hospital; Takeishi Yasuchika: Fukushima Medical University; Kazuomi Kario: Jichi Medical University School of Medicine.

(11 physicians, 8 institutes)

**Methods**

**Pulse transit time estimated blood pressure**

Pulse transit time (PTT) means travel time between the R-wave of the electrocardiography and the pulse wave at the site of the finger in plethysmography (Supplemental figure 1a) [1]. Arrival refers to the steepest part of the leading edge of the pulse wave. Pulse wave velocity was determined by PTT, height, and body correlation factor, as described previously [1, 2]. Then, the PTT-BP values were calculated automatically using DOMINO Light software version 1.5.0, based on the patented algorithm.

Gesche et al. reported the formula of PTT-estimated BP as follows. [1]

BP_PTT_ = *P*1 × PWV × e^(^*^P^*^3×PWV)^ + *P*2 × PWV*^P^*^4^ – (BP_PTT,cal_ – BP_cal_)

Here, *P1* = 700, *P2* = 766,000, *P3* = -1, and *P4* = 9. BP_PTT_ indicates blood pressure from PTT; BP_PTT,cal_, the calculated blood pressure (from PTT) corresponding to the blood pressure measured by the reference method; BP_cal_, blood pressure measured at a distinct time at the beginning of the experiment using the reference method, i.e., the cuff method. The parameters *P1-P4* were estimated by least square fitting of the function to the data of 13 subjects. More detailed information has been published by Gesche et al. [1]

Examples of PTT estimated systolic and diastolic BP measurements and portable sleep monitor are shown in supplemental figure 1b. These PTT estimated BP values is available to be extracted in the data set (supplemental figure 1c). Indexes of beat-to-beat PTT-estimated BP were used by all BP readings estimated by PTT (asterisk mark in supplemental figure 1c). Indexes of intermittent PTT-estimated BP were used by every 30 minutes (black arrow in supplemental figure 1c: This value is corresponded to BP value at AM 2:00.)

**References**

1. Gesche H, Grosskurth D, Kuchler G, Patzak A. Continuous blood pressure measurement by using the pulse transit time: comparison to a cuff-based method. Eur J Appl Physiol 2012; 112:309–315.
2. Davies JI, Struthers AD. Pulse wave analysis and pulse wave velocity: a critical review of their strengths and weaknesses. J Hypertens 2003; 21:463–472.

| **Supplemental Table 1. Comparison of pulse transit time (PTT)-estimated blood pressure parameters between beat-to-beat and intermittent measurement according to the presence and absence of AF** | | | | | | | | |
| --- | --- | --- | --- | --- | --- | --- | --- | --- |
|  | Without AF, n=214 | | |  | With AF, n=116 | | | P value  for difference |
|  | Beat-to-beat measurement | Intermittent  measurement | Difference |  | Beat-to-beat measurement | Intermittent  measurement | Difference |  |
| Average SBP, mmHg | 123.3±20.5 | 123.0±20.6 | 0.25^#^ |  | 120.3±19.0 | 120.2±19.1 | 0.15 | 0.457 |
| Average DBP, mmHg | 71.4±14.2 | 71.4±14.3 | 0.02 |  | 72.9±12.6 | 72.9±12.6 | -0.05 | 0.422 |
| Maximum SBP, mmHg | 153.6±27.1 | 133.6±21.7 | 20.0^‡^ |  | 146.6±23.9 | 129.4±20.1 | 17.2^‡^ | 0.050 |
| Maximum DBP, mmHg | 86.2±14.4 | 78.3±14.2 | 7.9^‡^ |  | 86.9±13.4 | 79.2±13.0 | 7.7^‡^ | 0.724 |
| Minimum SBP, mmHg | 106.6±19.6 | 114.4±20.5 | -7.8^‡^ |  | 105.6±18.1 | 112.4±18.3 | -6.8^‡^ | 0.150 |
| Minimum DBP, mmHg | 57.6±15.5 | 64.5±14.8 | -6.9^‡^ |  | 60.9±14.0 | 66.6±13.3 | -5.8^‡^ | 0.055 |
| SD of SBP, mmHg | 5.4±2.3 | 5.2±2.3 | 0.2^*^ |  | 4.7±1.6 | 4.5±1.6 | 0.2^*^ | 0.852 |
| SD of DBP, mmHg | 3.9±1.3 | 3.7±1.6 | 0.1^*^ |  | 3.4±1.2 | 3.3±1.2 | 0.1 | 0.518 |
| CV of SBP, % | 4.4±1.9 | 4.3±2.0 | 0.2^*^ |  | 4.0±1.4 | 3.8±1.3 | 0.2^#^ | 0.619 |
| CV of DBP, % | 5.8±3.0 | 5.6±3.2 | 0.2^*^ |  | 4.8±2.0 | 4.7±2.3 | 0.1 | 0.339 |
| ^*^P<0.05, ^#^P<0.01, ^‡^P<0.001 between groups. AF indicates atrial fibrillation; CV, coefficient variation; DBP, diastolic blood pressure; SBP, systolic blood pressure; SD, standard deviation. | | | | | | | | |

| **Supplemental Table 2. Comparison of pulse transit time (PTT)-estimated blood pressure parameters between beat-to-beat and intermittent measurement according to those with the use of calcium blocker and those without** | | | | | | | | |
| --- | --- | --- | --- | --- | --- | --- | --- | --- |
|  | Without the use of calcium blocker, n=208 | | |  | With the use of calcium blocker, n=122 | | | P value  for difference |
|  | Beat-to-beat measurement | Intermittent  measurement | Difference |  | Beat-to-beat measurement | Intermittent  measurement | Difference |  |
| Average SBP, mmHg | 118.0±18.7 | 117.8±18.8 | 0.22^#^ |  | 129.4±20.2 | 129.2±20.3 | 0.20 | 0.880 |
| Average DBP, mmHg | 71.6±13.6 | 71.6±13.7 | -0.03 |  | 72.5±13.8 | 72.5±13.9 | 0.02 | 0.586 |
| Maximum SBP, mmHg | 145.6±23.6 | 127.9±20.1 | 17.7^‡^ |  | 160.6±27.7 | 139.4±21.2 | 21.3^‡^ | 0.012 |
| Maximum DBP, mmHg | 86.0±13.8 | 78.3±13.8 | 7.7^‡^ |  | 87.2±14.5 | 79.2±13.9 | 8.0^‡^ | 0.591 |
| Minimum SBP, mmHg | 102.7±18.6 | 109.7±18.6 | -7.0^‡^ |  | 112.2±18.5 | 120.5±19.9 | -8.2^‡^ | 0.059 |
| Minimum DBP, mmHg | 59.3±14.8 | 65.1±14.2 | -5.8^‡^ |  | 57.9±15.5 | 65.5±14.7 | -7.6^‡^ | 0.001 |
| SD of SBP, mmHg | 5.0±2.0 | 4.9±2.0 | 0.2^*^ |  | 5.4±2.2 | 5.1±2.2 | 0.4^#^ | 0.141 |
| SD of DBP, mmHg | 3.6±1.3 | 3.5±1.5 | 0.1 |  | 3.8±1.4 | 3.7±1.4 | 0.1 | 0.647 |
| CV of SBP, % | 4.3±1.8 | 4.2±1.8 | 0.1^*^ |  | 4.2±1.7 | 4.0±1.7 | 0.3^#^ | 0.270 |
| CV of DBP, % | 5.3±2.4 | 5.2±2.8 | 0.1 |  | 5.5±3.4 | 5.4±3.1 | 0.2 | 0.461 |
| ^*^P<0.05, ^#^P<0.01, ^‡^P<0.001 between groups. CV indicates coefficient variation; DBP, diastolic blood pressure; SBP, systolic blood pressure; SD, standard deviation. | | | | | | | | |

| **Supplemental Table 3. Comparison of pulse transit time (PTT)-estimated blood pressure parameters between beat-to-beat and intermittent measurement according to those with the use of ARB and those without** | | | | | | | | |
| --- | --- | --- | --- | --- | --- | --- | --- | --- |
|  | Without the use of ARB, n=234 | | |  | With the use of ARB, n=96 | | | P value  for difference |
|  | Beat-to-beat measurement | Intermittent  measurement | Difference |  | Beat-to-beat measurement | Intermittent  measurement | Difference |  |
| Average SBP, mmHg | 120.2±20.5 | 120.0±20.7 | 0.19^*^ |  | 127.1±17.9 | 126.9±17.9 | 0.27^*^ | 0.576 |
| Average DBP, mmHg | 70.4±13.3 | 70.4±13.4 | -0.01 |  | 75.6±13.9 | 75.6±13.8 | -0.01 | 0.963 |
| Maximum SBP, mmHg | 149.2±26.7 | 130.3±21.9 | 18.9^‡^ |  | 156.0±24.4 | 136.6±18.8 | 19.4^‡^ | 0.731 |
| Maximum DBP, mmHg | 85.1±13.5 | 77.1±13.4 | 8.0^‡^ |  | 89.6±14.9 | 82.2±14.1 | 7.4^‡^ | 0.307 |
| Minimum SBP, mmHg | 104.5±20.1 | 111.7±20.3 | -7.2^‡^ |  | 110.5±15.8 | 118.4±17.4 | -8.0^‡^ | 0.281 |
| Minimum DBP, mmHg | 57.5±14.6 | 63.9±14.0 | -6.3^‡^ |  | 61.8±15.8 | 68.6±14.7 | -6.8^‡^ | 0.387 |
| SD of SBP, mmHg | 5.2±2.2 | 5.0±2.2 | 0.2^*^ |  | 5.2±1.7 | 4.9±1.8 | 0.3^#^ | 0.381 |
| SD of DBP, mmHg | 3.7±1.4 | 3.6±1.6 | 0.1^*^ |  | 3.6±1.1 | 3.5±1.2 | 0.1 | 0.524 |
| CV of SBP, % | 4.4±1.9 | 4.2±1.9 | 0.1^*^ |  | 4.1±1.4 | 3.9±1.5 | 0.2^#^ | 0.494 |
| CV of DBP, % | 5.6±3.0 | 5.5±3.2 | 0.2^*^ |  | 4.9±1.9 | 4.8±2.1 | 0.1 | 0.578 |
| ^*^P<0.05, ^#^P<0.01, ^‡^P<0.001 between groups. ARB indicates angiotensin II receptor blocker; CV, coefficient variation; DBP, diastolic blood pressure; SBP, systolic blood pressure; SD, standard deviation. | | | | | | | | |

| **Supplemental Table 4. Comparison of pulse transit time (PTT)-estimated blood pressure parameters between beat-to-beat and intermittent measurement according to those with the use of ACE inhibitor and those without** | | | | | | | | |
| --- | --- | --- | --- | --- | --- | --- | --- | --- |
|  | Without the use of ACE inhibitor, n=244 | | |  | With the use of ACE inhibitor, n=86 | | | P value  for difference |
|  | Beat-to-beat measurement | Intermittent  measurement | Difference |  | Beat-to-beat measurement | Intermittent  measurement | Difference |  |
| Average SBP, mmHg | 124.6±19.3 | 124.4±19.4 | 0.16^*^ |  | 115.5±20.6 | 115.1±20.8 | 0.38^‡^ | 0.128 |
| Average DBP, mmHg | 73.6±13.1 | 73.6±13.1 | -0.04 |  | 67.2±14.1 | 67.1±14.3 | 0.1 | 0.138 |
| Maximum SBP, mmHg | 153.9±25.8 | 134.6±20.4 | 19.3^‡^ |  | 143.4±25.7 | 125.2±22.0 | 18.2^‡^ | 0.478 |
| Maximum DBP, mmHg | 87.8±13.9 | 80.2±13.3 | 7.6^‡^ |  | 82.6±13.8 | 74.2±14.4 | 8.4^‡^ | 0.215 |
| Minimum SBP, mmHg | 107.9±18.3 | 116.0±19.2 | -8.1^‡^ |  | 101.5±20.5 | 107.1±20.0 | -5.6^‡^ | <0.001 |
| Minimum DBP, mmHg | 60.4±14.6 | 67.1±13.8 | -6.6^‡^ |  | 54.0±15.3 | 60.1±14.6 | -6.1^‡^ | 0.404 |
| SD of SBP, mmHg | 5.2±1.9 | 5.0±1.9 | 0.3^‡^ |  | 5.0±2.5 | 4.9±2.5 | 0.2 | 0.466 |
| SD of DBP, mmHg | 3.6±1.2 | 3.5±1.4 | 0.1^*^ |  | 4.0±1.4 | 3.9±1.6 | 0.1 | 0.835 |
| CV of SBP, % | 4.2±1.6 | 4.1±1.6 | 0.2^#^ |  | 4.4±2.1 | 4.3±2.2 | 0.1 | 0.664 |
| CV of DBP, % | 5.1±2.2 | 4.9±2.5 | 0.1^*^ |  | 6.4±3.8 | 6.2±3.7 | 0.2 | 0.664 |
| ^*^P<0.05, ^#^P<0.01, ^‡^P<0.001 between groups. ACE indicates angiotensin-converting enzyme; CV, coefficient variation; DBP, diastolic blood pressure; SBP, systolic blood pressure; SD, standard deviation. | | | | | | | | |

| **Supplemental Table 5. Comparison of pulse transit time (PTT)-estimated blood pressure parameters between beat-to-beat and intermittent measurement according to those with the use of diuretics and those without** | | | | | | | | |
| --- | --- | --- | --- | --- | --- | --- | --- | --- |
|  | Without the use of diuretics, n=217 | | |  | With the use of diuretics, n=113 | | | P value  for difference |
|  | Beat-to-beat measurement | Intermittent  measurement | Difference |  | Beat-to-beat measurement | Intermittent  measurement | Difference |  |
| Average SBP, mmHg | 124.9±19.8 | 124.6±20.0 | 0.24^#^ |  | 117.1±19.4 | 116.9±19.5 | 0.16 | 0.570 |
| Average DBP, mmHg | 74.4±13.3 | 74.3±13.4 | 0.03 |  | 67.2±13.2 | 67.3±13.3 | -0.1 | 0.176 |
| Maximum SBP, mmHg | 155.8±25.6 | 135.3±20.8 | 20.4^‡^ |  | 142.3±25.1 | 126.0±20.8 | 16.3^‡^ | 0.004 |
| Maximum DBP, mmHg | 88.8±13.8 | 80.8±13.2 | 8.1^‡^ |  | 81.9±13.5 | 74.5±14.0 | 7.4^‡^ | 0.213 |
| Minimum SBP, mmHg | 107.4±19.6 | 115.8±19.9 | -8.5^‡^ |  | 104.1±18.0 | 109.5±18.8 | -5.4^‡^ | <0.001 |
| Minimum DBP, mmHg | 61.3±14.7 | 67.8±14.1 | -6.5^‡^ |  | 53.9±14.5 | 60.4±13.5 | -6.5^‡^ | 0.940 |
| SD of SBP, mmHg | 5.5±2.2 | 5.2±2.1 | 0.3^‡^ |  | 4.5±1.7 | 4.5±1.9 | 0.01 | 0.012 |
| SD of DBP, mmHg | 3.6±1.3 | 3.5±1.4 | 0.1^*^ |  | 3.9±1.3 | 3.8±1.6 | 0.1 | 0.617 |
| CV of SBP, % | 4.5±1.9 | 4.3±1.8 | 0.3^‡^ |  | 3.9±1.5 | 3.9±1.7 | -0.01 | 0.011 |
| CV of DBP, % | 5.1±2.4 | 4.9±2.7 | 0.1 |  | 6.1±3.3 | 5.9±3.3 | 0.2 | 0.891 |
| ^*^P<0.05, ^#^P<0.01, ^‡^P<0.001 between groups. CV indicates coefficient variation; DBP, diastolic blood pressure; SBP, systolic blood pressure; SD, standard deviation. | | | | | | | | |

| **Supplemental Table 6. Comparison of pulse transit time (PTT)-estimated blood pressure parameters between beat-to-beat and intermittent measurement according to those with the use of beta-blocker and those without** | | | | | | | | |
| --- | --- | --- | --- | --- | --- | --- | --- | --- |
|  | Without the use of beta-blocker, n=146 | | |  | With the use of beta-blocker, n=184 | | | P value  for difference |
|  | Beat-to-beat measurement | Intermittent  measurement | Difference |  | Beat-to-beat measurement | Intermittent  measurement | Difference |  |
| Average SBP, mmHg | 125.2±20.4 | 125.1±20.5 | 0.13 |  | 119.9±19.4 | 119.6±19.5 | 0.29^‡^ | 0.226 |
| Average DBP, mmHg | 73.9±13.2 | 73.9±13.2 | -0.07 |  | 70.4±13.9 | 70.3±14.0 | 0.04 | 0.175 |
| Maximum SBP, mmHg | 157.3±27.8 | 135.8±21.5 | 21.5^‡^ |  | 146.3±23.7 | 129.2±20.5 | 17.1^‡^ | 0.001 |
| Maximum DBP, mmHg | 88.4±13.8 | 80.5±13.5 | 7.9^‡^ |  | 84.9±14.0 | 77.1±13.9 | 7.7^‡^ | 0.681 |
| Minimum SBP, mmHg | 107.2±19.3 | 116.3±20.3 | -9.1^‡^ |  | 105.4±19.0 | 111.6±19.1 | -6.1^‡^ | <0.001 |
| Minimum DBP, mmHg | 60.5±14.4 | 67.6±13.9 | -7.1^‡^ |  | 57.4±15.4 | 63.4±14.5 | -6.0^‡^ | 0.047 |
| SD of SBP, mmHg | 5.6±2.2 | 5.2±2.2 | 0.4^‡^ |  | 4.9±2.0 | 4.7±1.9 | 0.1 | 0.034 |
| SD of DBP, mmHg | 3.6±1.4 | 3.4±1.5 | 0.2^#^ |  | 3.7±1.3 | 3.7±1.5 | 0.04 | 0.094 |
| CV of SBP, % | 4.5±1.8 | 4.2±1.9 | 0.3^#^ |  | 4.1±1.8 | 4.0±1.7 | 0.1 | 0.056 |
| CV of DBP, % | 5.1±2.3 | 4.9±2.4 | 0.3^#^ |  | 5.7±3.1 | 5.6±3.2 | 0.1 | 0.138 |
| ^*^P<0.05, ^#^P<0.01, ^‡^P<0.001 between groups. CV indicates coefficient variation; DBP, diastolic blood pressure; SBP, systolic blood pressure; SD, standard deviation. | | | | | | | | |

| **Supplemental Table 7. Comparison of pulse transit time (PTT)-estimated diastolic blood pressure parameters between beat-to-beat and intermittent measurement according to tertiles of ODI** | | | | | |
| --- | --- | --- | --- | --- | --- |
|  | ODI/hr | | | P for trend | P_int_ |
|  | Tertile 1  (0-11.3)  n=110 | Tertile 2  (11.4-24.9)  n=110 | Tertile 3  (25.1-74.4)  n=110 |  |  |
| Average DBP, mmHg | | | | | |
| Beat-to-beat | 73.3±12.6 | 69.3±13.6 | 73.1±14.5 | 0.929 | 0.994 |
| Intermittent | 73.3±12.6 | 69.3±13.7 | 73.2±14.6 | 0.920 |  |
| Difference | -0.04±0.88 | 0.04±0.72 | -0.02±0.64 | 0.844 | NA |
| Maximum DBP, mmHg | | | | | |
| Beat-to-beat | 87.2±14.0 | 83.9±13.0 | 88.3±14.8 | 0.562 | 0.973 |
| Intermittent | 79.4±13.3 | 76.0±13.2 | 80.4±14.6 | 0.589 |  |
| Difference | 7.8±5.6^‡^ | 7.9±4.5^‡^ | 7.9±4.1^‡^ | 0.887 | NA |
| Minimum DBP, mmHg | | | | | |
| Beat-to-beat | 61.2±13.3 | 55.8±15.2 | 59.4±16.1 | 0.373 | 0.810 |
| Intermittent | 67.4±12.7 | 62.1±14.5 | 66.3±15.3 | 0.558 |  |
| Difference | -6.2±4.4^‡^ | -6.3±4.8^‡^ | -6.9±5.5^‡^ | 0.313 | NA |
| SD of DBP, mmHg | | | | | |
| Beat-to-beat | 3.4±1.3 | 3.8±1.3 | 3.9±1.3 | 0.008 | 0.751 |
| Intermittent | 3.2±1.5 | 3.7±1.5 | 3.8±1.4 | 0.006 |  |
| Difference | 0.20±0.80^*^ | 0.02±0.79 | 0.11±0.79 | 0.435 | NA |
| CV of DBP, % | | | | | |
| Beat-to-beat | 4.8±2.0 | 5.9±3.7 | 5.6±2.2 | 0.020 | 1.000 |
| Intermittent | 4.5±2.2 | 5.9±3.7 | 5.4±2.5 | 0.024 |  |
| Difference | 0.28±1.13^*^ | 0.04±1.37 | 0.14±1.14 | 0.397 | NA |
| P_int_ mean P for interaction between beat-to-beat and intermittent group. CV indicates coefficient of variation; DBP, diastolic blood pressure; SD, standard deviation; ODI, oxygen desaturation index; NA, not applicable. | | | | | |

**Supplemental Figure 1**


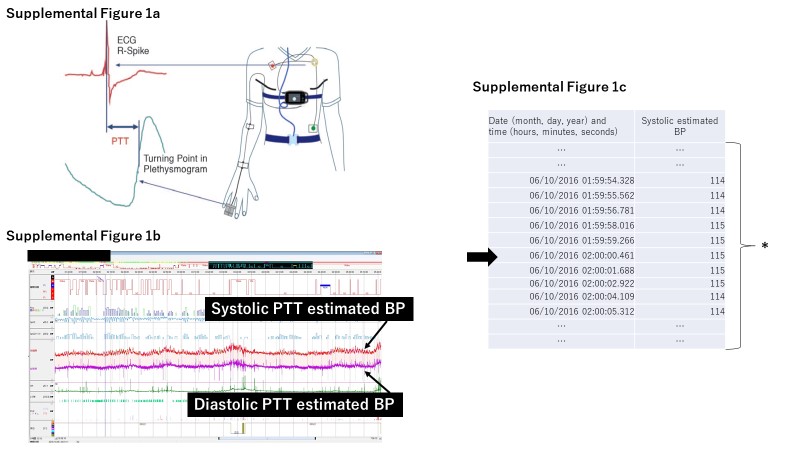


**Supplemental Figure 2**


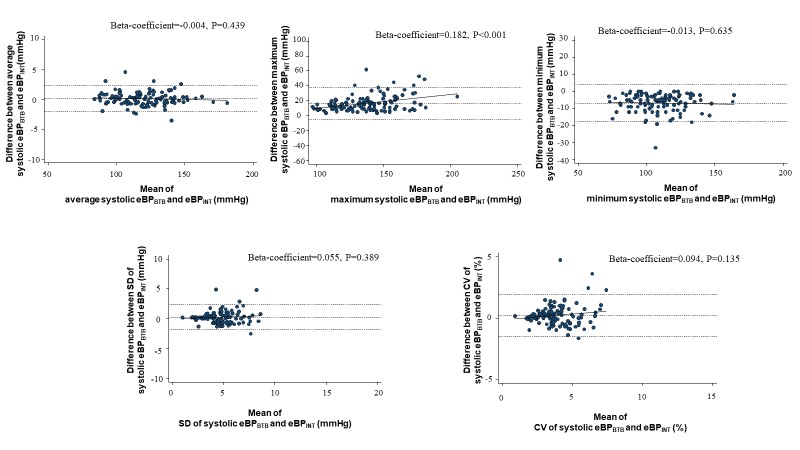


**Supplemental Figure 3**


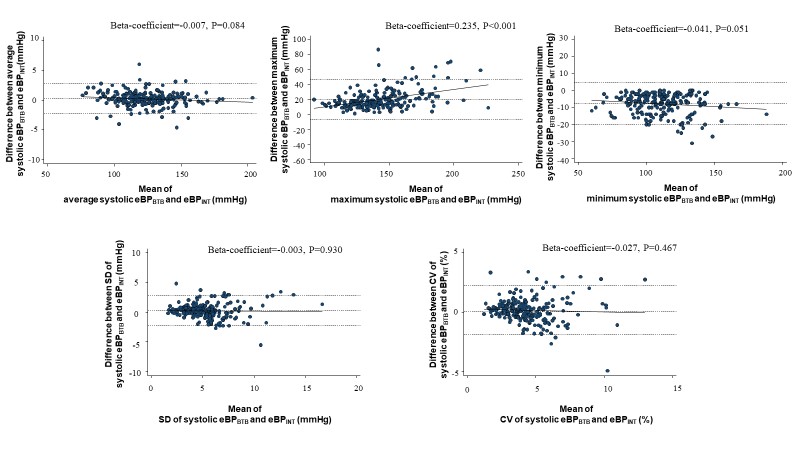


**Supplemental figure legends**

**Supplemental Figure 1**

The concept of pulse transit time (1a), representative BP estimations from an overnight portable monitor using pulse transit recording (1b), and extracted data (1c). As one example, the black arrow in supplemental Figure 1c is used as the intermittent PTT-estimated BP at fixed time intervals on AM 2:00. The data in the bracket marked with an asterisk are the beat-to-beat PTT-estimated BP values. BP indicates blood pressure; PTT, pulse transit time.

**Supplemental Figure 2**

Bland-Altman plots comparing the use of eBP_BTB_ and eBP_INT_ to assess systolic blood pressure parameters in patients with atrial fibrillation. eBP_BTB_ indicates beat-to-beat PTT-estimated BP; eBP_INT_, intermittent PTT-estimated BP at fixed time intervals.

**Supplemental Figure 3**

Bland-Altman plots comparing the use of eBP_BTB_ with eBP_INT_ to assess systolic blood pressure parameters in patients without atrial fibrillation. eBP_BTB_ indicates beat-to-beat PTT-estimated BP; eBP_INT_, intermittent PTT-estimated BP at fixed time intervals.
